# Student-Advising Recommendations from the Council of Residency Directors Student Advising Task Force

**DOI:** 10.5811/westjem.2016.10.31296

**Published:** 2016-11-21

**Authors:** Emily Hillman, Lucienne Lutfy-Clayton, Sameer Desai, Adam Kellogg, Xiao Chi Zhang, Kevin Hu, Jamie Hess

**Affiliations:** * Truman Medical Center, University of Missouri-Kansas City School of Medicine, Department of Emergency Medicine, Kansas City, Missouri; † Baystate Medical Center, University of Massachusetts Medical School-Baystate Health, Department of Emergency Medicine, Worcester, Massachusetts; ‡ University of Kentucky, Department of Emergency Medicine, Lexington, Kentucky; § Alpert Medical School of Brown University, Department of Emergency Medicine, Providence, Rhode Island; ¶ Icahn School of Medicine, Department of Emergency Medicine, New York, New York; || University of Wisconsin School of Medicine and Public Health, Department of Emergency Medicine, Madison, Wisconsin

## Abstract

Residency training in emergency medicine (EM) is highly sought after by U.S. allopathic medical school seniors; recently there has been a marked increase in the number of applications per student, raising costs for students and programs. Disseminating accurate advising information to applicants and programs could reduce excessive applying. Advising students applying to EM is a critical role for educators, clerkship directors, and program leaders (residency program director, associate and assistant program directors). A variety of advising resources is available through social media and individual organizations; however, currently there are no consensus recommendations that bridge these resources. The Council of Residency Directors (CORD) Student Advising Task Force (SATF) was initiated in 2013 to improve medical student advising. The SATF developed best-practice consensus recommendations and resources for student advising. Four documents (Medical Student Planner, EM Applicant’s Frequently Asked Questions, EM Applying Guide, and EM Medical Student Advisor Resource List) were developed and are intended to support prospective applicants and their advisors. The recommendations are designed for the mid-range EM applicant and will need to be tailored to students’ individual needs.

## BACKGROUND

Students considering applying to emergency medicine (EM) frequently look to medical school educators, EM faculty and residents, clerkship directors and program leaders (residency program director, associate and assistant program directors) for advising and mentorship. Advisors can help prepare students for a successful future by discussing topics such as individually-based career options, potential clinical experiences, and the application process.^1^,^2^ Effective advising is an acquired skill that necessitates careful consideration to help foster the student’s personal, professional, and educational growth while offering individualized guidance, with direct and honest answers to address the student’s anxieties and fears.^3^,^4^

One important factor in effective advising is knowledge of the issues specific to each student applying to an EM residency.^4^ Students considering a career in EM may lack access to faculty who can provide accurate advising information. Although advising is considered to be critically important, many prospective applicants seek an advisor late in their training or do not have an advisor before the application process.^5^,^6^ Students without access to local mentors may seek out “distance mentoring;” however, this requires that students first be aware of potential mentoring resources.^7^,^8^ The literature on mentoring in EM is scarce.^9^ While there is limited literature correlating measurable benefits of undergraduate mentoring, a recent study published in the *Western Journal of Emergency Medicine* found a positive relationship between match outcomes and perceived mentor effectiveness.^6^,^10^

In 2016, EM ranked as the third most commonly matched specialty for United States (U.S.) allopathic medical school seniors (U.S. senior) with fewer match-positions relative to internal medicine and pediatrics, the top two matched specialties.^11^ While the percentage of EM postgraduate year (PGY)-1 positions filled per U.S. senior has remained stable over the last five years^11^, U.S. seniors are applying to more programs.^12^,^13^ According to the National Resident Matching Program (NRMP) applicant survey, EM-matched U.S. seniors applied to an average of 26 programs in 2011 and 39 in 2015.^12^,^13^ During the same period, U.S. seniors who did not match applied to nearly twice as many programs (32 to 60), but received half as many interview offers (15 and 7).^12^,^13^ Though the overall competitiveness of EM has remained stable, increased applications have resulted in a heightened sense of EM competitiveness.

In 2013, during a Council of Residency Directors (CORD) Academic Assembly meeting, the Student Advising Task Force (SATF) was established to improve student advising. Variation in the quality and availability of student advising, as well as the increasing number of applications, led the task force to develop consensus documents to guide prospective EM applicants and their advisors. The task force formed as a joint venture with members of CORD, Clerkship Directors of EM (CDEM), the American Academy of Emergency Medicine (AAEM), and the Emergency Medicine Residents Association (EMRA). SATF members include faculty and residents representing programs throughout the country.

## OBJECTIVES

The goals of SATF in creating and disseminating consensus recommendations and advising documents are two-fold: 1) to provide advising resources and advice for students considering applying to EM; and 2) to equip faculty in advising roles with the knowledge and resources to provide high quality advising to students.

## CURRICULAR DESIGN

To identify best-practice advising information, SATF working groups were established; members self-selected to participate in working groups based on interest and expertise. Collation of available literature, existing advising resources, member opinion and experience guided the development of the consensus recommendations and documents. Group leaders worked between groups to ensure consistency between documents and to distribute materials to the task force as a whole for comments, revisions, and approval. The resources developed include the following:

1. **Medical Student Planner** – a chronological planner for each semester and year of medical school with recommendations for what to prioritize to maximize a student’s potential.
2. **EM Applicant’s Frequently Asked Questions (EM-FAQ)** – a brief question-and-answer guide that addresses the most commonly asked questions from applicants.
3. **EM Applying Guide** – a comprehensive document that provides in-depth answers on a broad range of topics, including planning visiting rotations, obtaining letters of recommendation, preparing an ERAS application, and navigating the interview and ranking process.
4. **EM Medical Student Advisor Resource List** – a comprehensive list of available high quality advising resources, including embedded links.

The CORD-SATF developed the following recommendations as best practice for student advising. These recommendations can be found within the aforementioned resources and were approved by the CORD Board of Directors, as well as by AAEM and CDEM. The recommendations are intended to serve as a general guide as each student needs an individualized approach.

1. **Pre-Clinical Years**: Students with an early interest in EM should be encouraged to consider how early academic achievement, volunteer activities, and career exposure can positively impact their ability to match in EM. Students should aim to be in the top half of their class in basic science courses. Consistent longitudinal volunteer experiences are valued. Research is not required for EM applicants to match but is considered a strength. Joining an EM interest group (EMIG) can help solidify the student’s career choice and open the opportunity for mentorship and research opportunities. For students at institutions without an interest group or EM faculty advisors, it would be especially beneficial to consider joining EMRA. EMRA can provide resident mentorship opportunities and advising resources.
2. **Emergency Medicine Rotations**: **Doing** two rotations in EM at institutions with training programs is recommended to allow for a variety of experiences, development of EM skills, and multiple perspectives on performance. A third rotation may be appropriate for some students depending on prior academic performance and application goals. Optimal timing is during the summer and fall months of a student’s fourth year. Most students at an institution with an academic EM program will do one rotation at their home school and a visiting rotation at another program. Students should consider participating in rotations that expose them to different practice varieties, locations, and program design. Students who excel in their rotations come prepared to work hard, are enthusiastic, develop EM presentations skills, create full management plans, and read to expand their medical knowledge.
3. **The Role of the Standardized Letter of Evaluation (SLOE)**: Letters of evaluation from within the specialty of EM are highly important factors in selecting applicants to interview, rivaling the importance of United States Medical Licensing Exam (USMLE) performance.^14^ Obtaining two SLOEs is recommended, preferably one from each EM rotation at a training program. These letters, often written by the education team, can provide a meaningful comparison group, and are considered less biased than other letters.
4. **USMLE Step 1 and Step 2 Clinical Knowledge (CK) Performance**: Each program will weigh test scores differently in their applicant review process. With a USMLE Step 1 or Step 2 CK score > 230 many programs will grant interviews.^14^ Students with a Step 1 score < 220 should be encouraged to take Step 2 CK early, to allow for results to be included in their initial application review.
5. **Electronic Residency Application Service (ERAS) Application**: It is recommended that students submit their application as early as possible, ideally on September 15 when ERAS opens. The personal statement is an area for the student to set himself apart and explain his interest, dedication and aptitude for EM. It is also an opportunity to address any discrepancies, delays, or perceived deficiencies in the student’s training and application. There is no standard number of applications that will guarantee matching in EM. Applicants should apply to a variety of programs across a spectrum of perceived competitiveness. Typical range for an applicant is 20–30 programs. Applications to > 40 programs is rarely warranted and often leads to diminishing returns. The number of applications to be made is particularly individualized and is best discussed directly with an EM advisor.
6. **Interviews**: Interviews at 10–12 programs correlates with a very high match rate.^14^ Students who are couples matching may need more interviews to reach a similar match rate. Independent applicants and those with “red flags” have a lower match rate overall but can still successfully match with fewer interviews. Once a student has decided he/she will not attend an interview, he or she should immediately cancel; a minimum of two weeks is recommended so the program can fill the interview spot.
7. **Rank List**: Students should rank programs based on their order of preference, not based on where they think they will appear on a program’s rank list. Other important factors to consider are location, program type, and the student’s personal experience.

Developing consensus documents to tackle the needs of advisors and the breadth of EM applicants met with multiple challenges:

1. **Applicant uniqueness**: No resource can meet all of the needs and questions of an individual applicant. Our resources represent consensus best-practice advice but are generalized to the average applicant. They do not supply the scalability applicants require to maximize their individual application. Students are encouraged to meet with individual advisors; when no advisor is available these resources can serve as a starting point. Over the next year our task force will produce addenda to better guide specific groups of applicants.
2. **Pre-existing resources**: In addition to SATF member expertise and opinion, the consensus recommendations and resources were developed by reviewing existing resources from the NMRP, CORD, EMRA, CDEM and AAEM, as well as blogs and social media. While these resources were developed using the best current advice, they are also largely based on opinion, remain subject to prejudices, and are inherently biased by their sources.
3. **Dissemination of materials**: Currently the resources are available on AAEM, CORD, CDEM and EMRA websites, and will be propagated via social media. Additionally, SATF resources and recommendations will need refinement and continuous revision.
4. **Lack of published data**: While associations can be inferred, there is no research on the success of applicants based on the advising received. Given the lack of evidence-based studies, the consensus recommendations are limited in that they are based on reviews of pre-existing resources, opinions, experience, and unanimity of the members participating in the SATF.

## IMPACT/EFFECTIVENESS

The CORD-SATF developed four resources through consensus recommendations to improve the advising of EM applicants and simultaneously support their advisors. These resources are endorsed by CORD, CDEM and AAEM and will be disseminated via multiple avenues. They will also be used to support distance advising for students without access to advising locally. These resources form a foundation for students and advisors to better understand the application process.

Time and continued application will reveal if the development of consensus advising recommendations improves the application experience for stakeholders. The SATF looks forward to the upcoming application cycles and NRMP data to evaluate the impact these resources have.

## References

[1] Garmel G (2004). Mentoring Medical Students in Academic Emergency Medicine. Acad Emerg Med.

[2] Zerzan JT, Hess R, Schur E (2009). Making the Most of Mentors: A Guide for Mentees. Acad Med.

[3] Bynny R (2012). Mentoring and coaching in medicine. The Pharos.

[4] Sambunjak D, Straus SE, Marusic A (2009). A Systematic Review of Qualitative Research on the Meaning and Characteristics of Mentoring in Academic Medicine. J Gen Intern Med.

[5] Sambunjak D, Straus SE, Marusic A (2006). Mentoring in academic medicine: a systematic review. JAMA.

[6] Dehon E, Cruse M, Dawson B (2015). Mentoring during Medical School and Match Outcome among Emergency Medicine Residents. West J Emerg Med.

[7] EMRA Hangouts. Emergency Medicine Residents’ Association Web Site.

[8] EMRA Student-Resident Mentorship Program. Emergency Medicine Residents’ Association Web Site.

[9] Yeung M, Nuth J, Stiell IG (2010). Mentoring in emergency medicine: the art and the evidence. CJEM.

[10] Frei E, Stamm M, Buddeberg-fischer B (2010). Mentoring programs for medical students – a review of the PubMed literature 2000–2008. BMC Med Educ.

[11] (2016). National Resident Matching Program, Results and Data: 2016 Main Residency Match^®^.

[12] National Resident Matching Program, Data Release and Research Committee (2011). Results of the 2011 NRMP Applicant Survey by Preferred Specialty and Applicant Type.

[13] National Resident Matching Program, Data Release and Research Committee (2015). Results of the 2015 NRMP Applicant Survey by Preferred Specialty and Applicant Type.

[14] National Resident Matching Program, Data Release and Research Committee (2014). Results of the 2014 NRMP Program Director Survey.

